# Targeting MUC1-C suppresses polycomb repressive complex 1 in multiple myeloma

**DOI:** 10.18632/oncotarget.20144

**Published:** 2017-08-10

**Authors:** Ashujit Tagde, Tahireh Markert, Hasan Rajabi, Masayuki Hiraki, Maroof Alam, Audrey Bouillez, David Avigan, Kenneth Anderson, Donald Kufe

**Affiliations:** ^1^ Dana-Farber Cancer Institute, Beth Israel Deaconess Medical Center, Harvard Medical School, Boston, MA, USA

**Keywords:** MUC1-C, multiple myeloma, BMI1, RING1, RING2

## Abstract

The polycomb repressive complex 1 (PRC1) includes the BMI1, RING1 and RING2 proteins. BMI1 is required for survival of multiple myeloma (MM) cells. The MUC1-C oncoprotein is aberrantly expressed by MM cells, activates MYC and is also necessary for MM cell survival. The present studies show that targeting MUC1-C with (i) stable and inducible silencing and CRISPR/Cas9 editing and (ii) the pharmacologic inhibitor GO-203, which blocks MUC1-C function, downregulates BMI1, RING1 and RING2 expression. The results demonstrate that MUC1-C drives *BMI1* transcription by a MYC-dependent mechanism. MUC1-C thus promotes MYC occupancy on the *BMI1* promoter and thereby activates BMI1 expression. We also show that the MUC1-C→MYC pathway induces RING2 expression. Moreover, in contrast to BMI1 and RING2, we found that MUC1-C drives RING1 by an NF-κB p65-dependent mechanism. Targeting MUC1-C and thereby the suppression of these key PRC1 proteins was associated with downregulation of the PRC1 E3 ligase activity as evidenced by decreases in ubiquitylation of histone H2A. Targeting MUC1-C also resulted in activation of the PRC1-repressed tumor suppressor genes, *PTEN, CDNK2A* and *BIM*. These findings identify a heretofore unrecognized role for MUC1-C in the epigenetic regulation of MM cells.

## INTRODUCTION

Mucin 1 (MUC1) is a cell membrane heterodimeric complex that is aberrantly expressed in primary multiple myeloma (MM) cells and MM cell lines [1-5]. The transmembrane MUC1 C-terminal (MUC1-C) subunit of the heterodimer functions as an oncoprotein that is necessary for the proliferation and survival of MM cells [5-8]. In this respect, the MUC1-C cytoplasmic domain is an intrinsically disordered 72-amino acid structure that has the plasticity to act as a node and intersect with multiple signaling pathways linked to self-renewal, inflammation and transformation [9, 10]. MUC1-C activates the proinflammatory IKK → NF-κB pathway, binds directly to NF-κB p65 and promotes the induction of NF-κB target genes, including *MUC1* itself in an autoinductive loop [11-13]. The MUC1-C cytoplasmic domain also binds directly to β-catenin, inhibits β-catenin degradation and activates the WNT/β-catenin/TCF4 pathway [14, 15]. Studies in MM cells have demonstrated that MUC1-C increases occupancy of β-catenin on the *MYC* promoter and drives *MYC* transcription [8]. Moreover, analysis of microarray datasets from primary MM cells showed that MUC1 expression positively correlates with that of MYC [8]. By extension, silencing MUC1-C in MM cells results in downregulation of MYC and thereby MYC target genes [8]. MM cells are addicted to MYC [16-18]. Thus, targeting MUC1-C with the downregulation of MYC explains, at least in part, why MM cells are dependent on MUC1-C for their proliferation and survival [5-8]. Of potential importance for targeting MUC1-C as a treatment for MM, the MUC1-C cytoplasmic domain includes a CQC motif that is essential for MUC1-C homodimerization, nuclear localization and function [6-8]. For these reasons, a cell-penetrating peptide, designated GO-203, has been developed that targets the MUC1-C CQC motif, inhibits MUC1-C homodimerization, nuclear import and function, and is effective in inducing MM cell death [6-8].

The polycomb repressive complex 1 (PRC1) includes the ring domain-containing BMI1, RING1 and RING2 proteins [19, 20]. BMI1 and RING1 bind to the catalytic RING2 subunit, and both contribute to the RING2 ubiquitin E3 ligase function [21]. BMI1 is also necessary for maintaining integrity of the complex [22]. In the prevailing hierarchical model, PRC1 is recruited to sites of H3K27 trimethylation (H3K27me3) generated by the polycomb repressive complex 2 (PRC2) [23], which includes enhancer of zeste homolog 2 (EZH2) and suppressor of zeste 12 homolog (SUZ12) components [24]. In turn, PRC1 catalyzes the mono-ubiquitination of histone H2A on K119 and promotes repression of *homeobox (HOX)* genes, among others [19-22, 25]. Of the PRC1 subunits, BMI1 has been linked to the self-renewal of normal stem cells and the tumorigenic potential of cancer stem-like cells (CSCs) [26-31]. BMI1 contributes to self-renewal and stemness by repressing the *CDNK2A* locus, which encodes the p16^INK4a^ and p14^ARF^ tumor suppressors [26, 28]. In carcinoma cells, BMI1 has also been linked to downregulation of the PTEN tumor suppressor [28, 32, 33]. Additionally, in MM cells, BMI1 suppresses the expression of multiple proapoptotic proteins, including BIM, and in this way is essential for MM self-renewal [34]. BMI1 also activates the WNT pathway by repressing the Dickkopf (DKK) family of WNT inhibitors [35]. Repression of DKK proteins contributes to activation of the *MYC* gene and thereby a positive-feedback loop linking the WNT pathway to induction of *BMI1* expression [35]. BMI1 is thus a potentially important target for the treatment of MM; however, to date, there are no clinically available BMI1 inhibitors [29]. Therefore, targeting upstream effectors that drive expression of BMI1 and other PRC1 components represents an attractive approach for reprogramming PRC1-mediated gene repression in MM.

The present studies demonstrate that MUC1-C drives expression of BMI1, RING1 and RING2 in MM cells. We show that MUC1-C activates the *BMI1* promoter by a MYC-mediated mechanism. In addition, we report that MUC1-C induces (i) RING2 by MYC-dependent signaling, and (ii) RING1 by activation of the NF-κB p65 pathway. In concert with these findings, targeting MUC1-C results in suppression of H2A ubiquitylation and induction of the PTEN, p14^ARF^ and BIM tumor suppressors in MM cells.

## RESULTS

### MUC1-C induces BMI1 expression in MM cells

**Figure 1:**
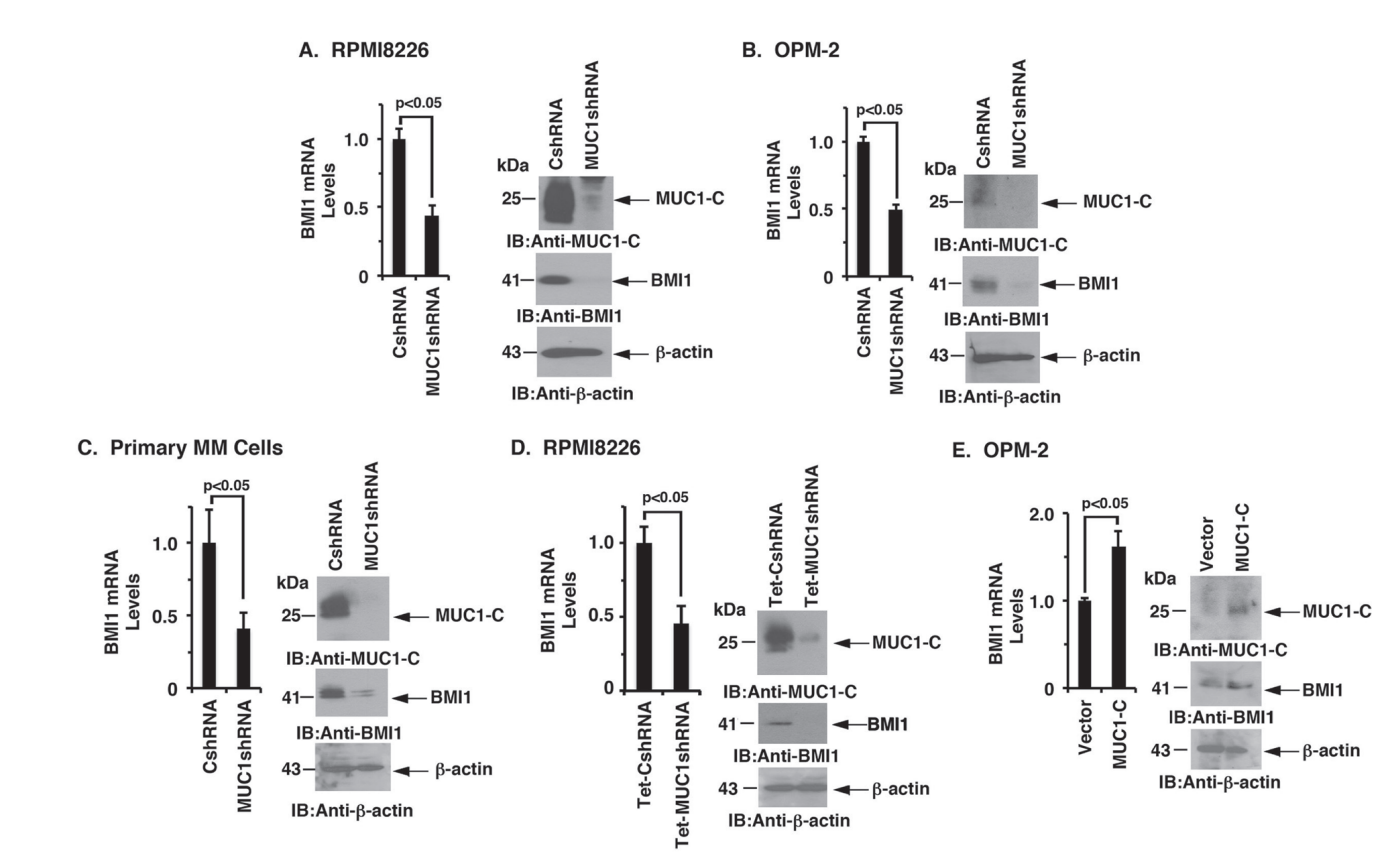
Targeting MUC1-C downregulates BMI1 expression **A.**-**C.** RPMI8226 (A), OPM-2 (B), and primary MM (C) cells from Patient #1 stably expressing a control shRNA (CshRNA) or a MUC1 shRNA were analyzed for BMI1 mRNA levels by qRT-PCR (left). The results (mean±SD of 3 determinations) are expressed as relative mRNA levels as compared with that obtained for the CshRNA cells (assigned a value of 1). Lysates were immunoblotted with the indicated antibodies (right). **D.** RPMI8226 cells were stably transduced to express a Tetracycline-inducible control shRNA (Tet-CshRNA) or a MUC1shRNA (Tet-MUC1shRNA). Cells treated with 200 ng/ml DOX for 5 d were analyzed for BMI1 mRNA levels by qRT-PCR (left). The results (mean±SD of 3 determinations) are expressed as relative mRNA levels compared with that obtained for control DOX-treated Tet-CshRNA cells (assigned a value of 1). Lysates were immunoblotted with the indicated antibodies (right). **E.** OPM-2 cells transiently transduced to express an empty vector or one expressing MUC1-C were analyzed for BMI1 mRNA levels by qRT-PCR (left). The results (mean±SD of 3 determinations) are expressed as relative mRNA levels as compared with that obtained for the vector cells (assigned a value of 1). Lysates from were immunoblotted with the indicated antibodies (right).

MUC1-C was stably silenced in MM cells to investigate whether this oncoprotein is involved in the regulation of BMI1 expression. In studies of RPMI8226 cells, MUC1-C silencing was associated with substantial downregulation of BMI1 mRNA and protein (Figure 1A, left and right). Similarly, silencing MUC1-C in OPM-2, KMS-12-E and U266 cells resulted in marked decreases in BMI1 expression (Figure 1B, left and right; Supplementary Figures 1A and 1B). In extending these observations to primary MM cells from 2 patients with refractory disease, we also found that targeting MUC1-C significantly reduces BMI1 levels (Figure 1C, left and right; Supplementary Figure 1C). To confirm these results, we stably transduced RPMI8226 cells to express a control Tet-CshRNA or a Tet-MUC1shRNA. DOX treatment of RPMI8226/Tet-MUC1shRNA, but not RPMI8226/Tet-CshRNA, cells was associated with significant downregulation of BMI1 mRNA and protein (Figure 1D, left and right). In concert with these results, the enforced overexpression of MUC1-C in OPM-2 cells was associated with an increase in BMI1 expression (Figure 1E, left and right), clearly indicating that MUC1-C and not the shed MUC1-N subunit is responsible for driving BMI1 expression.

**Figure 2:**
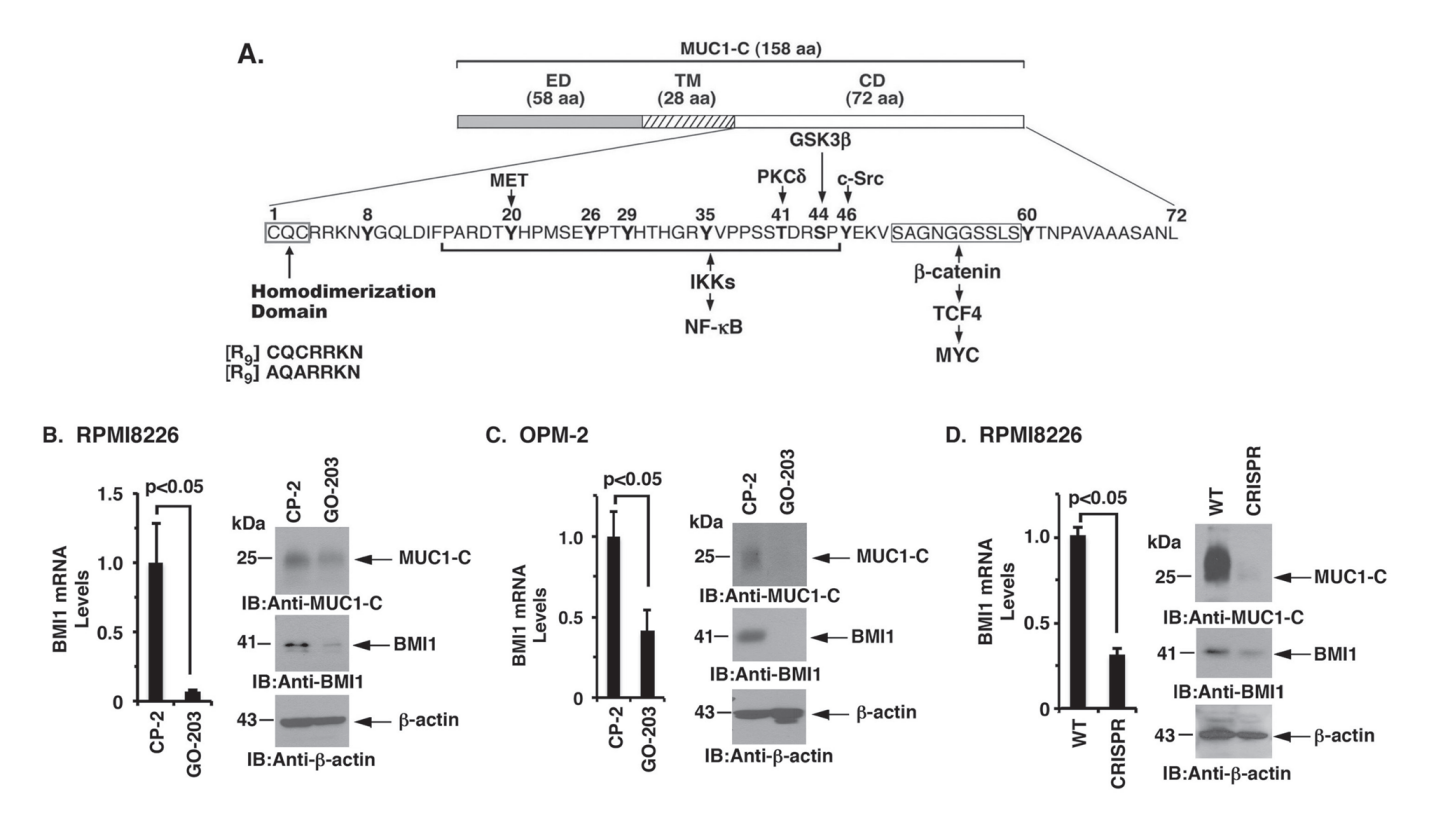
Targeting the MUC1-C cytoplasmic domain downregulates BMI1 expression **A.** Schema of the MUC1-C subunit with the 58-aa extracellular domain (ED), the 28-aa transmembrane domain (TM) and the 72-aa cytoplasmic domain (CD). Highlighted is the CQC motif, which is necessary and sufficient for MUC1-C homodimerization, and is targeted by GO-203 and not the control peptide CP-2. Also highlighted are interactions of the MUC1-C cytoplasmic domain with effectors of the NF-κB and MYC pathways. **B.**-**C.** RPMI8226 (B) and OPM-2 (C) cells were treated with 5 μM CP-2 or GO-203 for 48 h. The cells were analyzed for BMI1 mRNA levels by qRT-PCR (left). The results (mean±SD of 3 determinations) are expressed as relative mRNA levels as compared with that obtained for the CP-2-treated cells (assigned a value of 1). Lysates were immunoblotted with the indicated antibodies (right). **D.** RPMI8226 cells were silenced for MUC1 using CRISPR/Cas9 gene editing. The parental WT and CRISPR cells were analyzed for BMI1 mRNA levels by qRT-PCR (left). The results (mean±SD of 3 determinations) are expressed as relative mRNA levels as compared with that obtained for the WT cells (assigned a value of 1). Lysates were immunoblotted with the indicated antibodies (right).

The MUC1-C subunit consists of a 72-amino acid (aa) cytoplasmic domain with a CQC motif that is necessary and sufficient for the formation of MUC1-C homodimers and for MUC1-C-mediated transformation (Figure 2A). Accordingly, the cell-penetrating peptide inhibitor, GO-203, was developed to block the MUC1-C CQC motif in MM and other cancer cells (Figure 2A). Notably, treatment of RPMI8226 and OPM-2 cells with GO-203, but not the control peptide CP-2, induced a marked decrease in BMI1 mRNA and protein (Figure 2B and 2C, left and right). Downregulation of BMI1 expression was also observed in studies of GO-203-treated KMS-12-PE and NCIH929 cells (Supplementary Figure 2A and 2B). Furthermore, targeting MUC1-C with CRISPR/Cas9 gene editing was associated with a marked reduction in the BMI1 expression (Figure 2D, left and right; Supplementary Figure 2C). These findings collectively indicated that MUC1-C activates BMI1 expression in MM cells.

### MUC1-C drives *BMI1* transcription by a MYC-dependent mechanism

**Figure 3:**
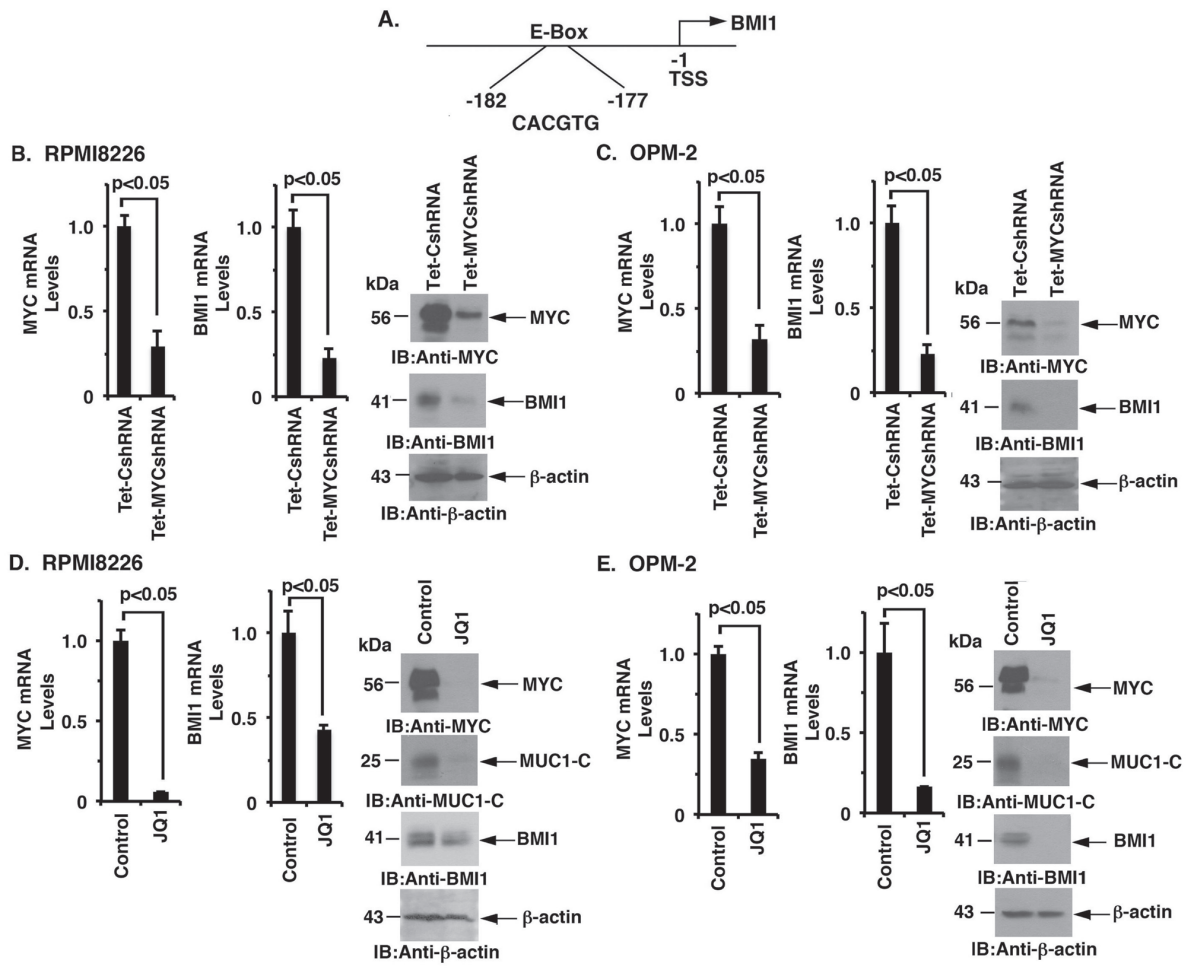
MUC1-C regulates BMI1 expression by a MYC-dependent mechanism **A.** Schema of the BMI1 promoter with highlighting of a MYC binding site (CACGTG) at positions -177 to -182. **B.**-**C.** RPMI8226/Tet-CshRNA and RPMI8226/Tet-MYCshRNA (B) and OPM-2/Tet-CshRNA and OPM-2/Tet-MYCshRNA (C) cells treated with DOX for 5 d were analyzed for MYC and BMI1 mRNA levels by qRT-PCR (left and middle). The results (mean±SD of 3 determinations) are expressed as relative mRNA levels compared with that obtained for the Tet-CshRNA cells (assigned a value of 1). Lysates were immunoblotted with the indicated antibodies (right). **D.**-**E.** RPMI8226 (D) and OPM-2 (E) cells treated with JQ1 or vehicle control for 48 h were analyzed for MYC and BMI1 mRNA levels by qRT-PCR (left and middle). The results (mean±SD of 3 determinations) are expressed as relative mRNA levels compared with that obtained for the control cells (assigned a value of 1). Lysates were immunoblotted with the indicated antibodies (right).

MUC1-C induces *MYC* expression in MM cells by activating the WNT/β-catenin/TCF-4 pathway [8]. The *BMI1* promoter contains a consensus E-Box (CACGTG) at position -177 to -182 upstream to the transcription start site (Figure 3A), invoking the possibility that MUC1-C induces BMI1 expression through the MYC pathway in MM cells. To address this potential mechanism, we assessed the effects of targeting MYC on BMI1 expression. RPMI8226 cells were thus transduced to stably express a Tet-inducible MYC shRNA (Tet-MYCshRNA) or a Tet-inducible control shRNA (Tet-CshRNA). Treatment of RPMI8226/Tet-MYCshRNA cells with DOX resulted in the marked suppression of MYC and BMI1 levels as compared to that in DOX-treated RPMI8226/Tet-CshRNA cells (Figure 3B, left, middle and right). These results were confirmed in DOX-treated OPM-2/Tet-CshRNA and OPM-2/Tet-MYCshRNA cells (Figure 3C, left, middle and right). In extending these observations, we found that treatment of RPMI8226 cells with the BET bromodomain inhibitor, JQ1, was associated with the marked suppression of MYC, MUC1-C and BMI1 expression (Figure 3D, left, middle and right). Similar results were obtained in studies of JQ1-treated OPM-2 cells (Figure 3E, left, middle and right), supporting the notion that the MUC1-C → MYC pathway induces BMI1 expression in MM cells.

### MUC1-C activates the *BMI1* promoter by enhancing MYC occupancy

**Figure 4:**
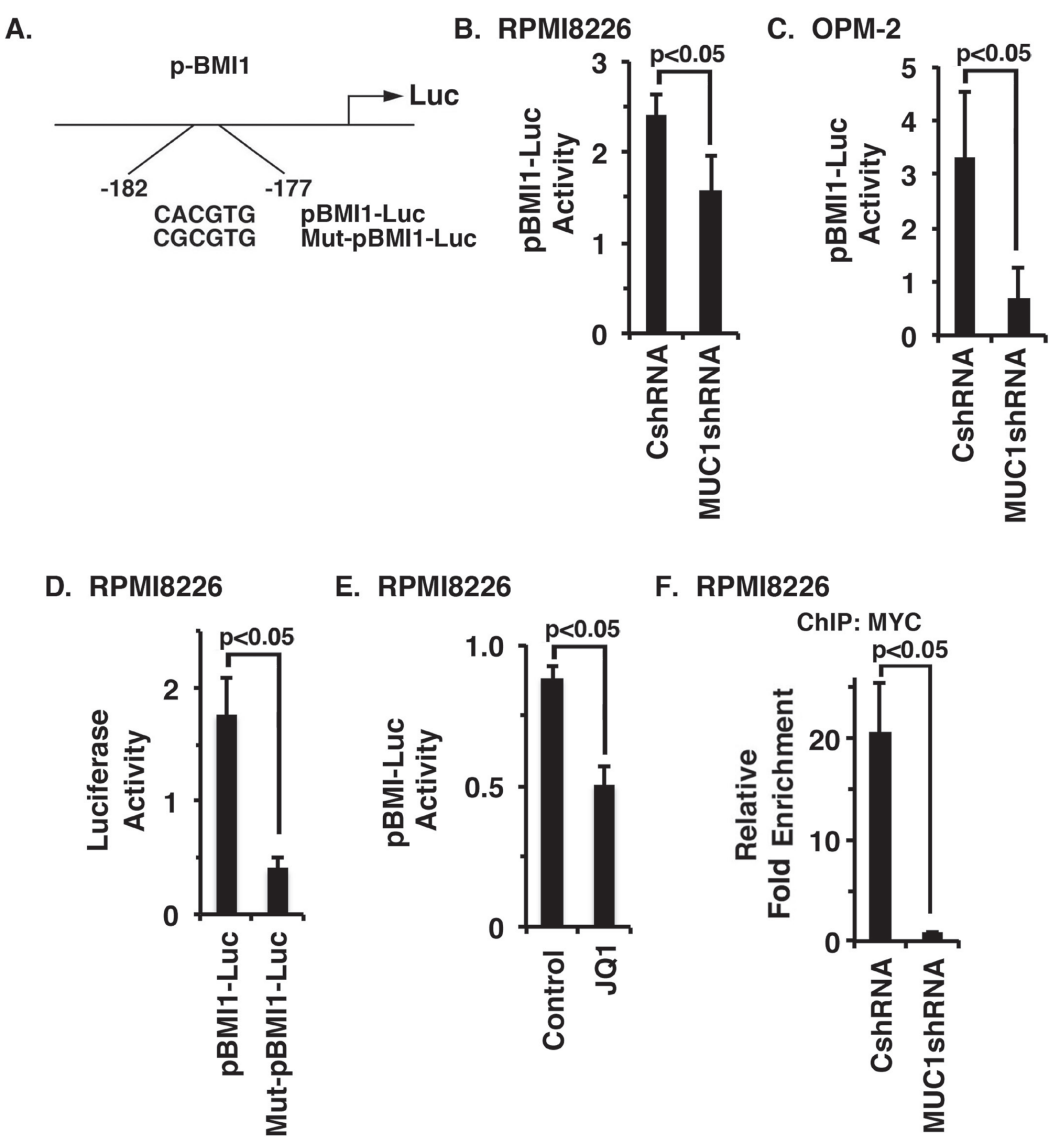
MUC1-C activates BMI1 gene transcription by a MYC-dependent mechanism **A.** Schema of the pBMI1-Luc reporter with highlighting of the wild-type (WT) and mutated MYC binding site. **B.**-**C.** The designated RPMI8226 (B) and OPM-2 (C) cells expressing a CshRNA or MUC1shRNA were transfected with pGL3-Luc or pBMI1-Luc and SV40-Renilla-Luc as an internal control. Luciferase activity was measured at 24 h after transfection. The results (mean±SE of 3 determinations) are expressed as the relative luciferase activity compared with that obtained with cells expressing pGL3-Luc. **D.** RPMI8226 cells were transfected with (i) pGL3-Luc, (ii) pBMI1-Luc or Mut-pBMI1-Luc and (iii) SV40-Renilla-Luc. Luciferase activity was measured at 24 h after transfection. The results (mean±SE of 3 determinations) are expressed as the relative luciferase activity compared with that obtained with cells expressing pGL3-Luc. **E.** RPMI8226 cells were treated with JQ1 or vehicle control for 48 h and then transfected with the pGL3-Luc or pBMI1-Luc and SV-40-Renilla-Luc. Luciferase activity was measured at 24 h after transfection. The results (mean±SE of 3 determinations) are expressed as the relative luciferase activity compared with that obtained with cells expressing pGL3-Luc. **F.** Soluble chromatin from the RPMI8226/CshRNA and RPMI8226/MUC1shRNA cells was precipitated with anti-MYC or a control IgG antibody. The final DNA samples were amplified by qPCR with pairs of primers (Supplementary Table 2) for the MYC binding site in the *BMI1* promoter. The results (mean±SE of 3 determinations) are expressed as the relative fold enrichment compared with that obtained for the IgG control (assigned a value of 1).

To determine whether MUC1-C drives *BMI1* transcription by a MYC-mediated mechanism, we transfected MM cells to express a pBMI1-Luc reporter that includes the MYC binding site (Figure 4A). Using this approach, we found that activation of pBMI1-Luc is decreased in RPMI8226/MUC1shRNA, as compared to RPMI8226/CshRNA, cells (Figure 4B). Silencing MUC1-C in OPM-2 cells was also associated with suppression of pBMI1-Luc activity (Figure 4C). Mutation of the MYC binding site (CACGTG→CGCGTG) in Mut-pBMI1-Luc (Figure 4A) resulted in downregulation of reporter activity (Figure 4D; Supplementary Figure 3A). Moreover, JQ1 treatment of RPMI8226 and OPM-2 cells significantly reduced activation of the pBMI1-Luc reporter (Figure 4E; Supplementary Figure S3B). In concert with involvement of the MUC1-C → MYC pathway, ChIP analysis of the *BMI1* promoter in RPMI8226 (Figure 4F) and OPM-2 (Supplementary Figure 3C) cells demonstrated that silencing MUC1-C is associated with decreases in MYC occupancy.

### MUC1-C induces RING1 and RING2 expression

**Figure 5:**
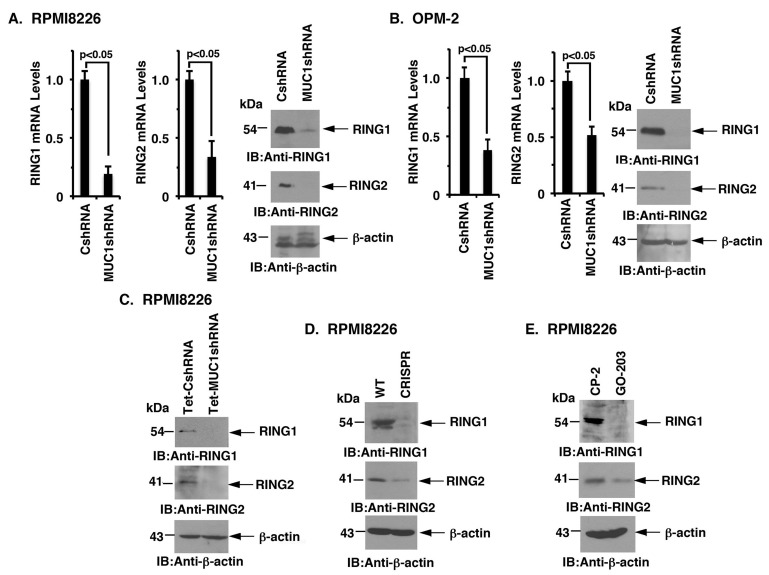
Targeting MUC1-C downregulates RING1 and RING2 expression **A.**-**B.** The designated RPMI8226 (A) and OPM-2 (B) cells expressing a CshRNA or MUC1shRNA were analyzed for RING1 and RING2 mRNA levels by qRT-PCR (left and middle). The results (mean±SD of 3 determinations) are expressed as relative mRNA levels as compared with that obtained for the CshRNA cells (assigned a value of 1). Lysates were immunoblotted with the indicated antibodies (right). **C.**-**E.** Lysates from DOX-treated RPMI8226/Tet-CshRNA and RPMI8226/Tet-MUC1shRNA (C), RPMI8226/WT and RPMI8226/CRISPR (D), and CP-2- and GO-203-treated RPMI8226 cells (E) were immunoblotted with the indicated antibodies.

PRC1 catalytic activity is dependent on BMI1, RING1 and RING2 [19-21]. Interestingly and as found for BMI1, targeting MUC1-C significantly reduced RING1 and RING2 levels in RPMI8226 (Figure 5A) and OPM-2 (Figure 5B), KMS-12-PE (Supplementary Figure 4A, left and right), U266 (Supplementary Figure 4B, left and right) and NCIH929 (Supplementary Figure S4C) cells. Targeting MUC1-C with (i) DOX-inducible silencing (Figure 5C), (ii) CRISPR/Cas9 gene editing (Figure 5D), and (iii) pharmacologic inhibition with GO-203 (Figure 5E) was also associated with the downregulation of both RING1 and RING2. Additionally, overexpression of MUC1-C in OPM-2 cells was associated with upregulation of RING1 and RING2 expression (Supplementary Figure 4D, indicating that, like BMI1, MUC1-C drives RING1 and RING2 expression.

### MUC1-C differentially activates RING1 and RING2

**Figure 6:**
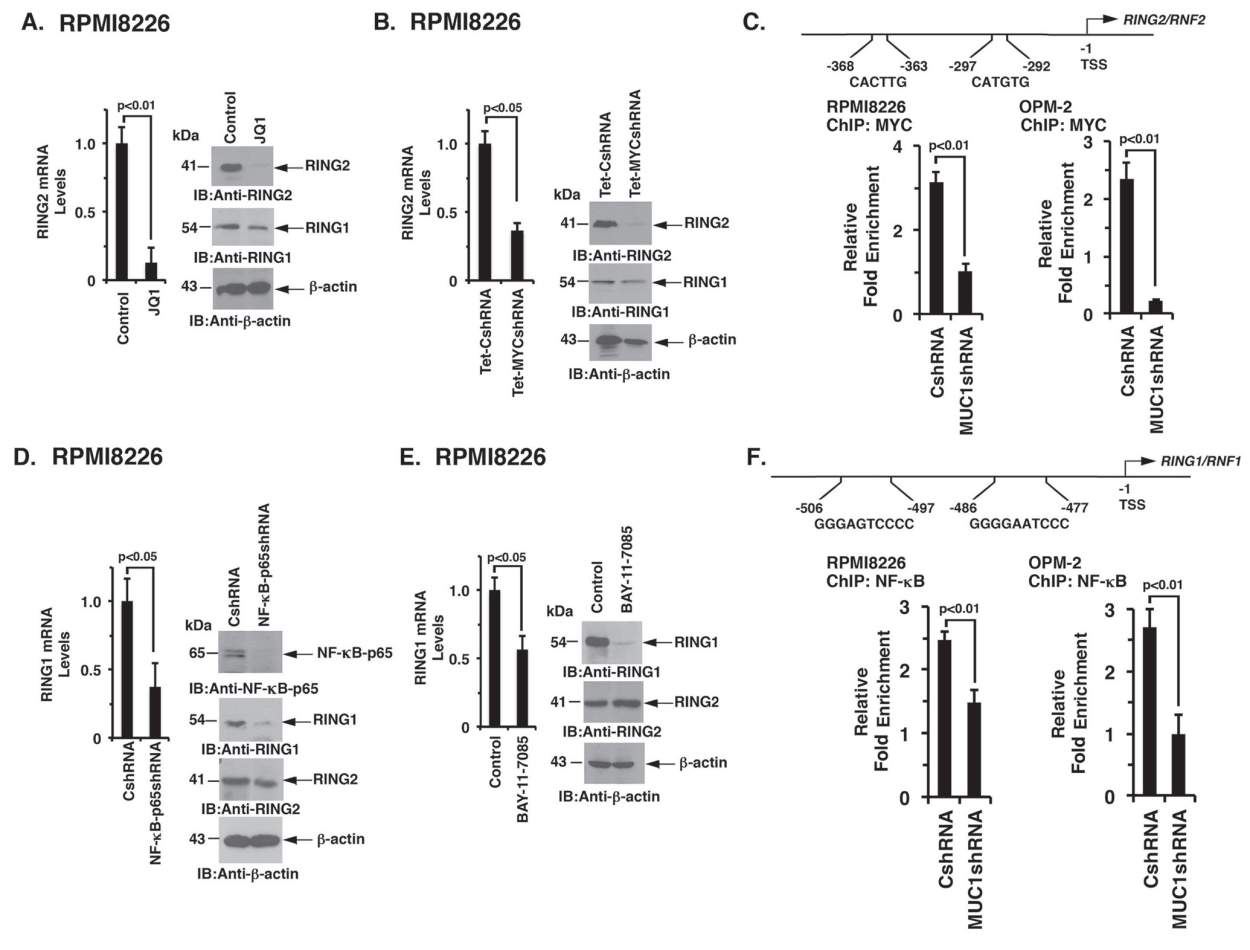
MUC1-C regulates RING2 and RING1 by MYC- and NF-κB p65-dependent mechanisms, respectively **A.** RPMI8226 cells treated with JQ1 or vehicle control for 48 h were analyzed for RING2 mRNA levels by qRT-PCR (left). The results (mean±SD of 3 determinations) are expressed as relative mRNA levels as compared with that obtained for the vehicle treated cells (assigned a value of 1, left). Lysates were immunoblotted with the indicated antibodies (right). **B.** RPMI8226/Tet-CshRNA and RPMI8226/Tet-MYCshRNA cells treated with DOX for 5 d were analyzed for RING2 mRNA levels by qRT-PCR (left). The results (mean±SD of 3 determinations) are expressed as relative mRNA levels as compared with that obtained for the Tet-CshRNA cells (assigned a value of 1). Lysates were immunoblotted with the indicated antibodies (right). **C.** Schema of the *RING2* promoter with highlighting of putative MYC binding sites. Soluble chromatin from the RPMI8226/CshRNA and RPMI8226/MUC1shRNA (left) and OPM-2/CshRNA and OPM-2/MUC1shRNA (right) cells was precipitated with anti-MYC or a control IgG antibody. The final DNA samples were amplified by qPCR with pairs of primers (Supplementary Table S2) encompassing the MYC binding sites in the *RING2* promoter. The results (mean±SE of 3 determinations) are expressed as the relative fold enrichment compared with that obtained for the IgG control (assigned a value of 1). **D.** RPMI8226/CshRNA and RPMI8226/NF-κBshRNA were analyzed for RING1 mRNA levels by qRT-PCR (left). The results (mean±SD of 3 determinations) are expressed as relative mRNA levels as compared with that obtained for the CshRNA cells (assigned a value of 1). Lysates were immunoblotted with the indicated antibodies (right). **E.** RPMI8226 cells treated with the 5 μM BAY-11-7085 or vehicle control for 24 h were assessed for RING1 levels by qRT-PCR (left). The results (mean±SD of 3 determinations) are expressed as relative mRNA levels as compared with that obtained for the vehicle treated cells (assigned a value of 1). Lysates were immunoblotted with the indicated antibodies (right). **F.** Schema of the *RING1* promoter with highlighting of putative NF-κB p65 binding sites. Soluble chromatin from the RPMI8226/CshRNA and RPMI8226/MUC1shRNA (left) and OPM-2/CshRNA and OPM-2/MUC1shRNA (right) cells was precipitated with anti-NF-κB p65 or a control IgG antibody. The final DNA samples were amplified by qPCR with pairs of primers (Supplementary Table 2) encompassing the NF-κB p65 binding sites in the *RING1* promoter. The results (mean±SE of 3 determinations) are expressed as the relative fold enrichment compared with that obtained for the IgG control (assigned a value of 1).

Notably, little is known about the regulation of RING1 and RING2. However, based on the identification of a MUC1-C → MYC → BIM1 pathway, we asked if MUC1-C induces the expression of RING1 and RING2 by a similar mechanism. In this context, inhibiting MYC with JQ1 was associated with marked downregulation of RING2 and a modest decrease in RING1 expression in RPMI8226 (Figure 6A, left and right) and OPM-2 (Supplementary Figure 5A) cells. DOX-inducible silencing of MYC in RPMI8226 (Figure 6B, left and right) and OPM-2 (Supplementary Figure 5B) cells also decreased RING2, and to a lesser extent RING1, mRNA and protein levels. Notably, the *RING2,* but not the *RING1,* promoter contains putative E-boxes for potential MYC binding (Figure 6C). Moreover and in support of these findings, targeting MUC1-C decreased MYC occupancy on the *RING2* promoter (Figure 6C, left and right). MUC1-C also activates NF-κB p65 in association with epigenetic regulation [36]. In this regard, silencing NF-κB p65 in RPMI8226 (Figure 6D, left and right) and OPM-2 (Supplementary Figure 5C) cells was associated with significant decreases in RING1, but not RING2, mRNA and protein. Moreover, BAY-11-7085 treatment of RPMI8226 (Figure 6E, left and right) and OPM-2 (Supplementary Figure 5D) cells suppressed RING1 and not RING2 expression. Furthermore, analysis of *RING1* promoter revealed consensus sites for potential NF-κB p65 binding (Figure 6F). Targeting MUC1-C also decreased NF-κB p65 occupancy on the *RING1* promoter (Figure 6F, left and right). These results collectively supported a model in which MUC1-C drives (i) RING2 by a MYC-mediated mechanism and (ii) RING1 predominantly by the NF-κB p65 pathway.

### Targeting MUC1-C derepresses PTEN, p14^ARF^ and BIM expression

**Figure 7:**
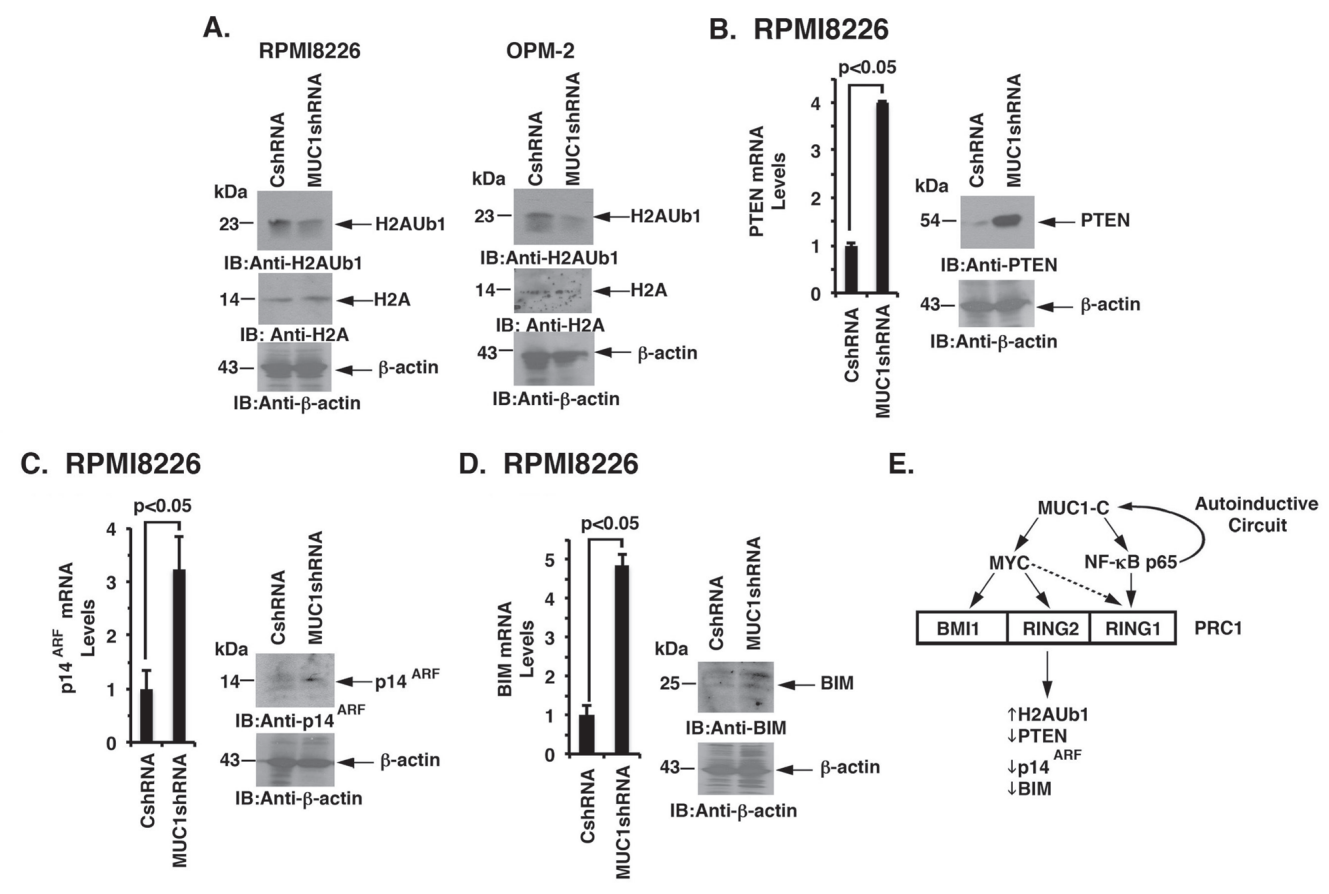
Targeting the MUC1-C → PRC1 pathway derepresses PTEN, p14ARF and BIM **A.** Lysates from the designated RPMI8226 (left) and OPM-2 (right) cells expressing a CshRNA or MUC1shRNA were immunoblotted with the indicated antibodies. **B.**-**D.** RPMI8226/CshRNA and RPMI8226/MUC1shRNA cells were analyzed for PTEN (B), p14^ARF^ (C) and BIM (D) mRNA levels by qRT-PCR (left). The results (mean±SD of 3 determinations) are expressed as relative mRNA levels as compared with that obtained for the CshRNA cells (assigned a value of 1). Lysates were immunoblotted with the indicated antibodies (right). **E.** Schema depicting the proposed involvement of MUC1-C in driving expression of the PRC1 components, BMI1, RING2 and RING1. MUC1-C activates the *MYC* gene in MM cells [8]. In turn, MYC occupies the *BMI1* promoter by a MUC1-C-dependent mechanism and induces BMI1 expression. Targeting MUC1-C and thereby MYC also resulted in the downregulation of RING2, supporting a similar pathway for the regulation of BMI1 and RING2. MUC1-C binds directly to NF-κB p65 and promotes activation of NF-κB p65-target genes, including *MUC1* itself in an autoinductive circuit [12]. Along these lines, targeting MUC1-C and NF-κB p65 resulted in the suppression of RING1 expression, indicating that MUC1-C regulates these three PRC1 components by a least two pathways. In contrast to BMI1 and RING2, targeting MYC was also associated with partial downregulation of RING1. In concert with the involvement of MUC1-C in regulating PRC1 components, the results further support a model in which MUC1-C activates PRC1 function and thereby the upregulation of H2AUb1 levels and suppression of PTEN, p14^ARF^ and BIM expression in MM cells.

PRC1 possesses H2A-K119 ubiquitin E3 ligase activity and catalyzes the ubiquitylation of H2A [32]. As expected from the suppression of BMI1, RING1 and RING2, we also found that targeting MUC1-C downregulates H2A ubiquitylation in RPMI8226 and OPM-2 cells (Figure 7A, left and right). PRC1 has been linked to the repression of certain tumor suppressor genes, such as *PTEN* and *INK4a/ARF* (*CDKN2A*), in carcinoma cells [26, 28, 32]. By extension, we found that silencing MUC1-C in RPMI8226 (Figure 7B, left and right) and OPM-2 (Supplementary Figure S6A) cells is associated with the derepression of PTEN expression. Targeting MUC1-C with CRISPR/Cas9 was also associated with significant upregulation of PTEN levels (Supplementary Figure 6B). In addition, targeting MUC1-C resulted in the induction of p14^ARF^ (Figure 7C, left and right; Supplementary Figure S6C). Other work has shown that BMI1 suppresses the proapoptotic BIM protein in MM cells [34]. Along these lines, silencing MUC1-C and thereby downregulation of BMI1 was associated with the upregulation of BIM (Figure 7D, left and right; Supplementary Figure S6D). These findings thus support a model in which (i) MUC1-C drives expression of BMI1, RING1 and RING2, and (ii) targeting MUC1-C suppresses PRC1 function in MM cells.

## DISCUSSION

The findings that MUC1-C is invariably expressed in MM cells [1-4] and is necessary for their growth and survival [6-8] have supported MUC1-C as a potential target for MM treatment. In addition, the recent demonstration that MUC1-C drives MYC in MM cells provided additional support for the importance of MUC1-C in the pathogenesis of MM [8]. In this respect, there is abundant evidence that MYC is essential for the progression and survival of MM cells [16-18, 37]. What was not clear, however, is how the MUC1-C → MYC pathway functions in promoting the malignant MM phenotype. The present studies show that MUC1-C induces expression of the BMI1 component of the PRC1 complex and that this response is mediated by a MYC-dependent mechanism. Notably, BMI1 was identified based on its role in accelerating Myc-induced lymphomagenesis [38] and has been linked to promoting self-renewal of cancer and leukemic stem cells [27, 30, 31]. In addition, BMI1 is overexpressed in MM cells [39-41] and is essential for their growth [38]. Our results show that MUC1-C promotes MYC occupancy on the *BMI1* promoter and thereby activates *BMI1* transcription in MM cells. In this way, targeting MUC1-C with (i) inducible and stable silencing, or (ii) the GO-203 inhibitor suppressed activation of the *BMI1* promoter and BMI1 expression. These findings do not exclude the possibility that MUC1-C regulates BMI1 by other mechanisms. For instance, studies in carcinoma cells have shown that MUC1-C induces miR-200c by an NF-κB p65-mediated signaling [42] and that miR-200c inhibits BMI1 translation [43, 44]. Recent work has further shown that MUC1-C forms a complex with BMI1 in cancer cells [44], invoking the possibility that MUC1-C also affects BMI1 expression by post-translational mechanisms.

PRC1 is formed by binding of BMI1 to RING1 and the active RING2 subunit, which catalyzes the ubiquitylation of H2A and promotes gene silencing [19-21]. Surprisingly, we found that targeting MUC1-C in MM cells is also associated with suppression of RING1 and RING2. Based on our findings with BMI1 and a lack of available information regarding the regulation of RING1 and RING2, we asked whether the MUC1-C → MYC pathway also drives expression of these additional PRC1 components. We found that, like BMI1, targeting MYC is associated with marked downregulation of RING2. In concert with a potential role for MUC1-C → NF-κB p65 signaling in regulating PRC1 function, we also found that targeting MUC1-C and NF-κB p65 suppresses RING1 expression. ChIP studies further demonstrated that MUC1-C promotes (i) MYC occupancy on the *RING2* promoter, and (ii) NF-κB p65 occupancy on the *RING1* promoter. Other studies will be needed to address the observation that targeting MYC is associated with partial downregulation of RING1 expression, which may be related to cross-talk between MUC1-C and MYC signaling. Our findings nonetheless underscore the notion that MUC1-C drives the expression of multiple components of the PRC1 complex, albeit by at least two distinct mechanisms, and thereby can integrate the regulation of BMI1, RING1 and RING2 in MM cells (Figure 7E). Our findings further support the premise that MUC1-C activates PRC1 function in epigenetic regulation. Indeed, targeting MUC1-C was associated with a marked decrease in PRC1-mediated H2A ubiquitylation and the induction of the PRC1-targeted tumor suppressor genes, *PTEN* and *INK4a/ARF* (Figure 7E). In concert with the demonstration that BIM1 suppresses the *BIM* gene in MM cells [34], we also found that targeting MUC1-C is associated with induction of BIM expression (Figure 7E). Our findings have thus identified a previously unrecognized role for MUC1-C in MM cells by activating the PRC1 complex and thereby the repression of certain TSGs.

With regard to translational relevance, PTC-209 is a small molecule inhibitor of BMI1 expression that has shown preclinical activity against colorectal and lung adenocarcinomas [29, 45]; however, to our knowledge, there are no clinically available BMI1 inhibitors. Our results indicate that targeting MUC1-C represents an alternative strategy for suppressing the expression of BMI1, as well as RING1 and RING2. In this regard, the MUC1-C inhibitor GO-203 has completed a Phase I trial in patients with advanced solid tumors and, based on the findings that GO-203 is highly synergistic with decitabine [46], is presently under study in a Phase II trial in combination with decitabine for patients with AML (NCT02204085). Therefore, the present results provide the basis for targeting MUC1-C and thereby reprogramming of the MM epigenome alone or in combination with other agents.

## MATERIALS AND METHODS

### Cell culture

RPMI8266, OPM-2, KMS-12-PE, U266 and NCIH929 (ATCC, Manassas, VA, USA) cells were cultured in RPMI1640 medium supplemented with 10% heat-inactivated fetal bovine serum (FBS), 100 U/mL penicillin, 100 μg/mL streptomycin and 2 mM L-glutamine. Primary CD138+ MM cells from 2 patients with refractory disease were isolated by Ficoll gradient separation using CD138 MicroBeads (Miltenyi Biotec, Somerville, MA, USA) and maintained in long-term culture in IMDM medium supplemented with 20% heat-inactivated FBS as described [8, 47, 48, 49]. Cells were treated with (i) doxycycline (DOX; Sigma, St. Louis, MO, USA), (ii) MUC1-C inhibitor GO-203 ([R]_9_-CQCRRKN) or inactive control peptide CP-2 ([R]_9_-AQARRKN), (iii) BRD4 inhibitor JQ1 [50], and (iv) NF-κB p65 inhibitor BAY-11-7085 (Santa Cruz Biotechnology, Dallas, TX, USA) or vehicle control dimethylsulfoxide (DMSO; Sigma).

### MUC1, MYC and NF-κB p65 silencing

MM cell lines and primary cells were infected with lentiviral vectors expressing a MUC1 shRNA, NF-κB p65 shRNA or scrambled control shRNA vector (Sigma) [8, 46]. Cells were selected and maintained in puromycin. For DOX-inducible MUC1 and MYC silencing, MUC1shRNA (MISSION shRNA; Sigma, TRCN0000122938), MYCshRNA (MISSION shRNA; Sigma, TRCN0000039642) or a control scrambled CshRNA (Sigma) was inserted into the pLKO-Tet-puro vector (Addgene, Cambridge, MA, USA, Plasmid #21915). Viral vectors were produced in HEK293T cells as described [51, 52]. Cells expressing Tet-shRNA vectors were selected in puromycin. MUC1 was also silenced with CRISPR/Cas9 gene editing as described [8].

### Real-time quantitative reverse-transcription PCR (qRT-PCR)

Total RNA was isolated using the RNAeasy Mini Kit (Invitrogen, Carlsbad, CA, USA). Complementary DNA (cDNA) was synthesized using the High Capacity cDNA Reverse Transcription Kit (Applied Biosystems, Foster City, CA, USA) [8]. The cDNA samples were amplified using the SYBR Green qRT-PCR assay kit and the ABI Prism 7000 sequence detector (Applied Biosystems). Primers used for qRT-PCR analysis are listed in Supplementary Table 1. The results were analyzed using the ΔΔ cycle threshold (DDCt) method as described [53]. Statistical significance was determined by the Student’s t test.

### Immunoblot analysis

Cells were lysed in NP-40 buffer containing protease inhibitor cocktail (ThermoScientific, Waltham, MA, USA). Immunoblotting was performed with anti-MUC1-C (NeoMarkers, Fremont, CA, USA), anti-BMI1, anti-RING1, anti-RING2, anti-H2AUb1, anti-H2A, anti-PTEN, anti-p14^ARF^ (Cell Signaling Technology, Danvers, MA, USA), anti-NF-κB p65, anti-BIM (Santa Cruz Biotechnology), anti-MYC (Abcam, Cambridge, MA, USA) and anti-β-actin (Sigma).

### Chromatin immunoprecipitation (ChIP) assays

Soluble chromatin was precipitated with anti-MYC (Abcam) or a control nonimmune IgG (Santa Cruz Biotechnology). For real-time ChIP quantitative polymerase chain reactions (qPCRs), the SYBR green system was used with the ABI Prism 7000 sequence detector (Applied Biosystems). Data are reported as relative fold enrichment as described [13, 36]. Primers used for qPCR are listed in Supplementary Table 2 [44].

### Promoter-reporter assays

Cells were transfected with an empty pGL3 luciferase (Luc) reporter vector, a pGL3-pBMI1-Luc promoter-reporter or a mutated Mut-pBMI1-Luc vector provided by Dr. Goberdhan Dimri [35, 44] and SV-40-*Renilla*-Luc in the presence of Lipofectamine™ 3000 Reagent (Invitrogen). After 24-48 h, the transfected cells were lysed using passive lysis buffer. The lysates were analyzed using the dual luciferase assay system (Promega, Madison, WI, USA).

## SUPPLEMENTARY MATERIALS FIGURES AND TABLES



## Abbreviations

MUC1: mucin 1
MUC1-C: MUC1 C-terminal subunit
MM: multiple myeloma
PcG: polycomb group proteins
PRC1: polycomb repressive complex 1
BMI1: B cell-specific Moloney murine leukemia virus integration site 1
RING: ring finger protein
PRC2: polycomb repressive complex 2
EZH2: enhancer of zeste homolog 2
SUZ12: suppressor of zeste 12 homolog
DNMT: DNA methyltransferase
TSG: tumor suppressor gene
DOX: doxycycline
