# Supplementary material for: Targeting MUC1-C suppresses polycomb repressive complex 1 in multiple myeloma
Source: Oncotarget. 2017 Aug 10;8(41):69237–49. doi: 10.18632/oncotarget.20144 (PMC5642475; doi:10.18632/oncotarget.20144)
Supplement: Supplementary file 1 [file oncotarget-08-69237-s001.pdf]

Targeting MUC1-C suppresses polycomb repressive complex 1 in multiple myeloma

SUPPLEMENTARY MATERIALS

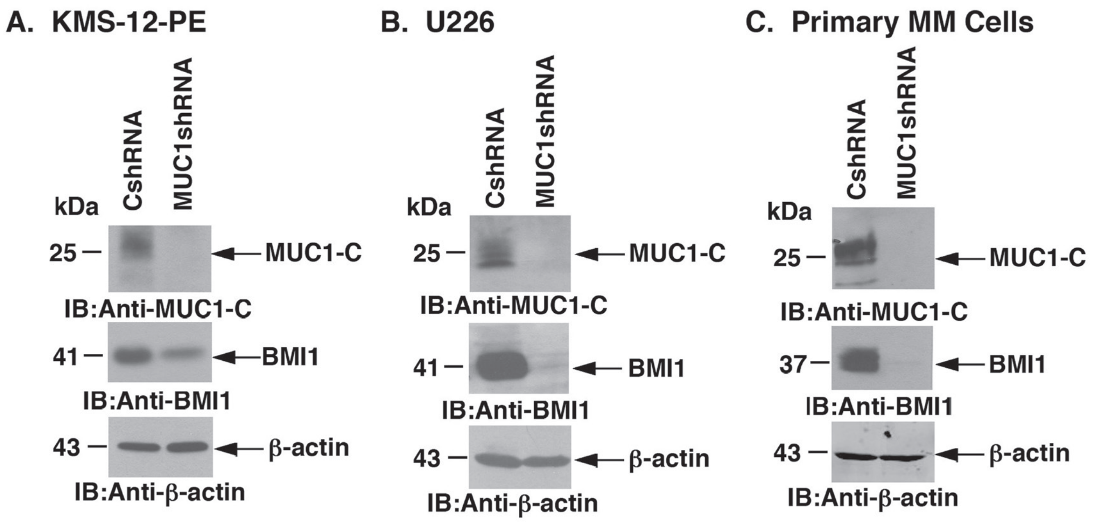

Supplementary Figure 1: Targeting MUC1-C is associated with the downregulation of BMI1 levels. (A-C) Lysates from KMS-12-PE (A), U266 (B) and primary MM (C) cells from Patient #2 stably expressing a control shRNA (CshRNA) or a MUC1 shRNA were immunoblotted with the indicated antibodies.

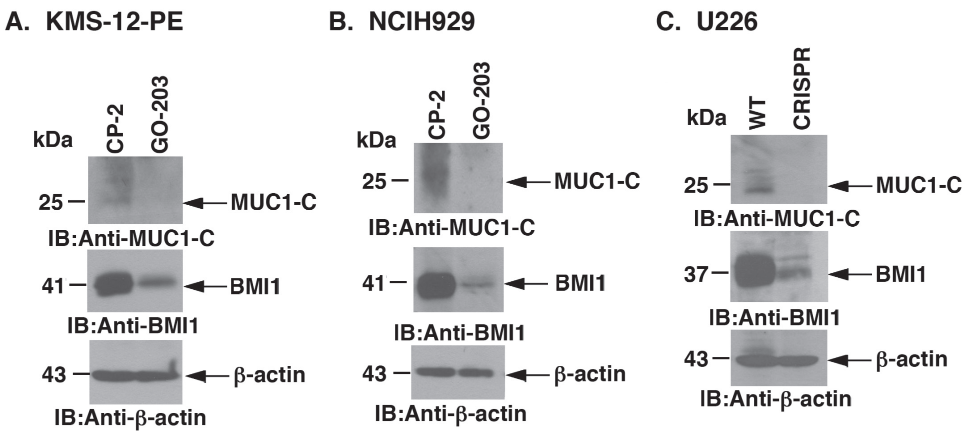

Supplementary Figure 2: MUC1-C drives BMI1 expression. (A-B) KMS-12-PE (A) and NCIH929 (B) cells were treated with CP-2 or GO-203 for 48 h. Lysates were immunoblotted with the indicated antibodies. (C) U266 cells were silenced for MUC1 using CRISPR/Cas9 gene editing. Lysates from the parental WT and CRISPR cells were analyzed by immunoblot analysis.

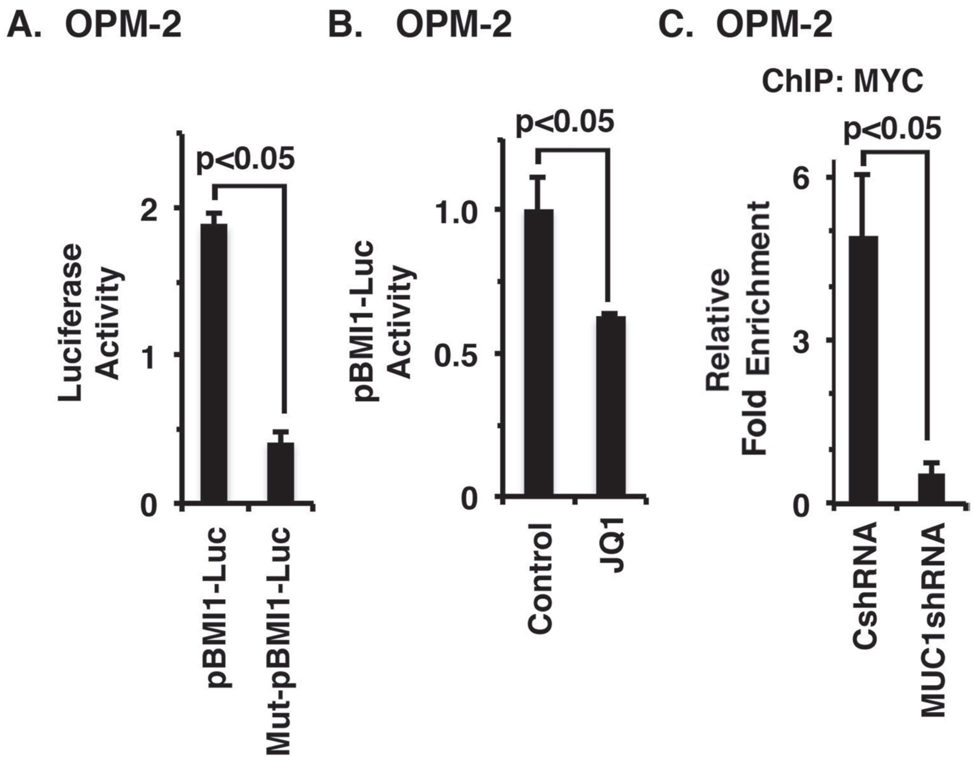

Supplementary Figure 3: MUC1-C activates BMI1 gene transcription. (A) OPM-2 cells were transfected with empty pGL3-Luc, the indicated pBMI1-Luc or a mutant Mut-pBMI1-Luc vectors and SV-40-Renilla-Luc. Luciferase activity was measured at 24 h after transfection. The results (mean±SE of 3 determinations) are expressed as the relative luciferase activity compared with that obtained with cells expressing pGL3-Luc. (B) OPM-2 cells were treated with JQ1 or vehicle control for 48 h and then transfected with the pGL3-Luc or pBMI1-Luc and SV-40-Renilla-Luc. Luciferase activity was measured at 24 h after transfection. The results (mean±SE of 3 determinations) are expressed as the relative luciferase activity compared with that obtained with cells expressing pGL3-Luc. (C) Soluble chromatin from the OPM-2/CshRNA and OPM-2/MUC1shRNA cells was precipitated with anti-MYC or a control IgG antibody. The final DNA samples were amplified by qPCR with pairs of primers (Supplemental Table S2) for the MYC binding site in the BMI1 promoter. The results (mean±SE of 3 determinations) are expressed as the relative fold enrichment compared with that obtained for the IgG control (assigned a value of 1).

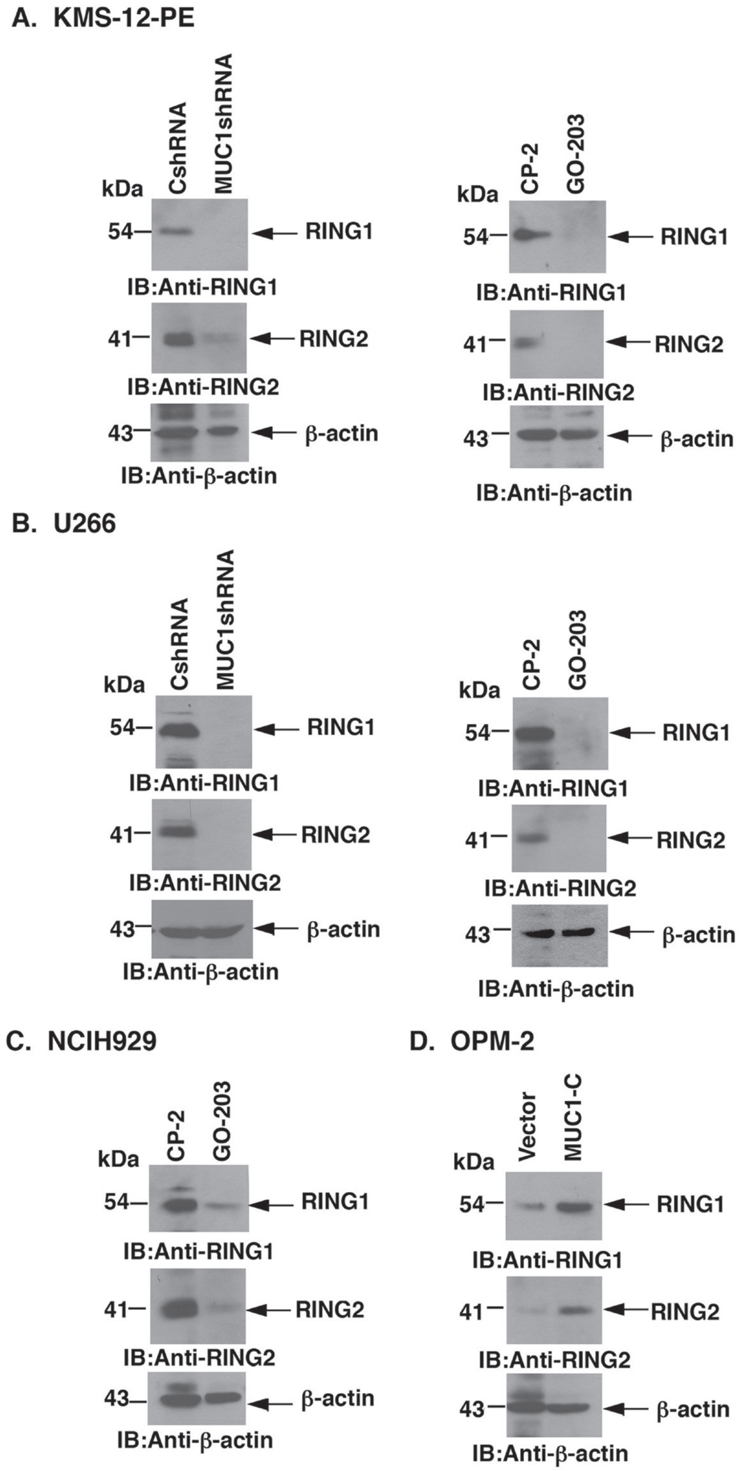

Supplementary Figure 4: Targeting MUC1-C was associated with the downregulation of RING1 and RING2 levels. (A-B) Lysates from KMS-12-PE (A) and U266 (B) cells stably expressing a control shRNA (CshRNA) or a MUC1 shRNA were immunoblotted with the indicated antibodies (left). Lysates from KMS-12-PE (A) and U266 (B) cells treated with CP-2 or GO-203 for 48 h were immunoblotted with the indicated antibodies (right). (C) Lysates from NCIH929 cells treated with CP-2 or GO-203 for 48 h were immunoblotted with the indicated antibodies. (D) OPM-2 cells transiently transduced to express an empty vector or one expressing MUC1-C were immunoblotted with the indicated antibodies.

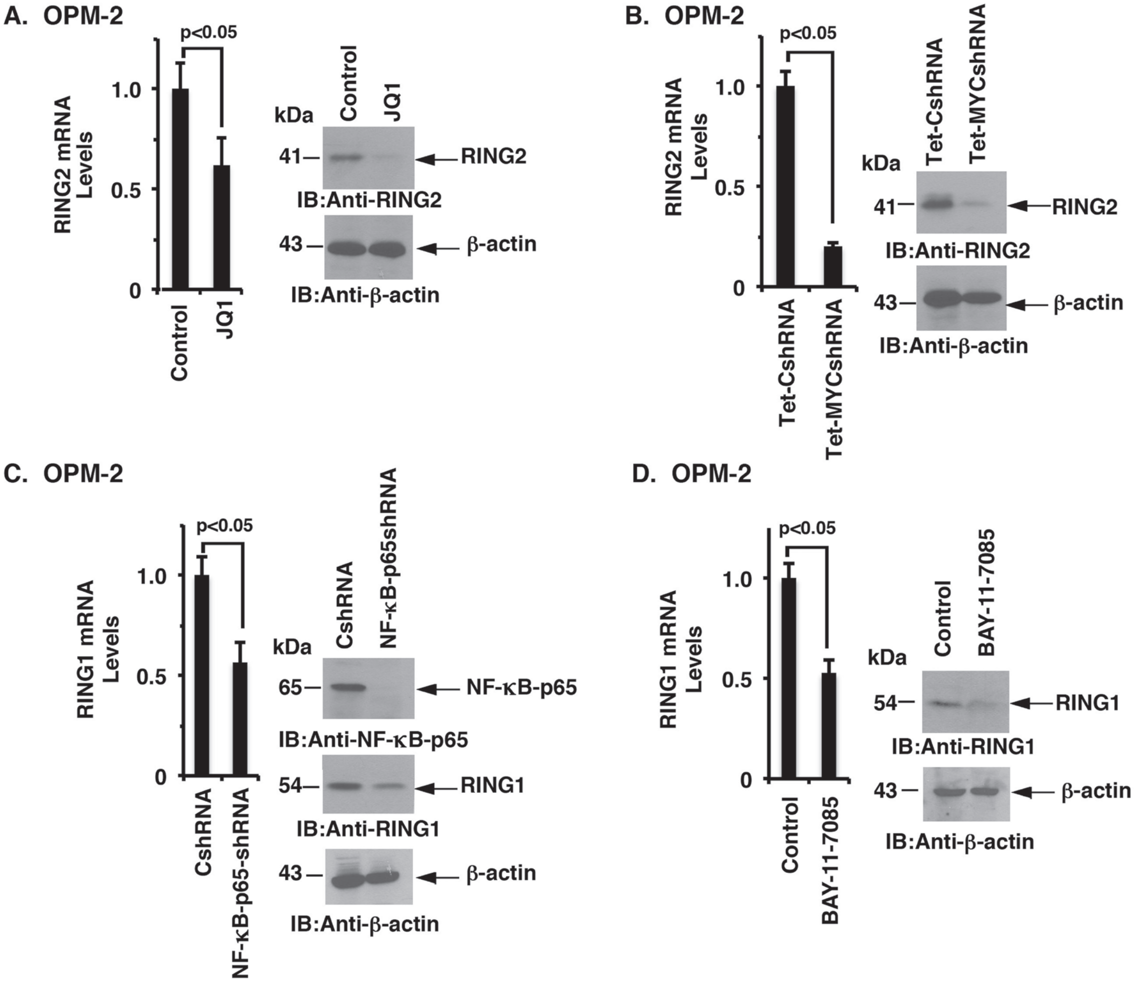

Supplementary Figure 5: MUC1-C induces RING1 and RING2 expression. (A) OPM-2 cells treated with JQ1 and vehicle control for 48 h were analyzed for RING2 mRNA levels by qRT-PCR (left). The results (mean±SD of 3 determinations) are expressed as relative mRNA levels as compared with that obtained for the control cells (assigned a value of 1). Lysates were immunoblotted with the indicated antibodies (right). (B) OPM-2/Tet-CshRNA and OPM-2/Tet-MYCshRNA cells treated with DOX for 5 d were analyzed for RING2 mRNA levels by qRT-PCR (left). The results (mean±SD of 3 determinations) are expressed as relative mRNA levels as compared with that obtained for the Tet-CshRNA cells (assigned a value of 1). Lysates were immunoblotted with the indicated antibodies (right). (C) OPM-2/CshRNA and OPM-2/NF-κB p65shRNA cells were analyzed for RING1 mRNA levels by qRT-PCR (left). The results (mean±SD of 3 determinations) are expressed as relative mRNA levels as compared with that obtained for the CshRNA cells (assigned a value of 1). Lysates were immunoblotted with the indicated antibodies (right). (D) OPM-2 cells treated with BAY-11-7085 or vehicle control for 24 h were analyzed for RING1 mRNA levels by qRT-PCR (left). The results (mean±SD of 3 determinations) are expressed as relative mRNA levels as compared with that obtained for the control cells (assigned a value of 1). Lysates were immunoblotted with the indicated antibodies (right).

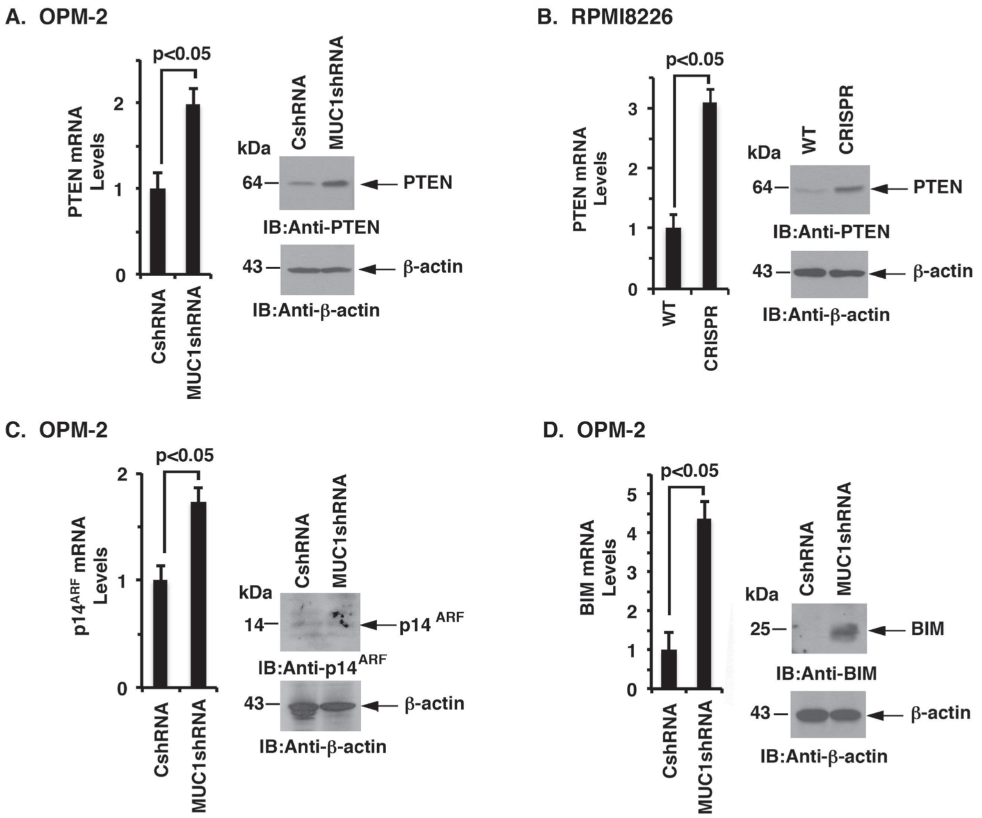

Supplementary Figure 6: Targeting MUC1-C induces PTEN, p14ARF and BIM expression. (A-D) The indicated cells were analyzed for mRNA levels by qRT-PCR (left). The results (mean±SD of 3 determinations) are expressed as relative mRNA levels as compared with that obtained for control cells (assigned a value of 1). Lysates were immunoblotted with the indicated antibodies (right).

Supplementary Table 1: Primers used for qRT-PCR.

MYC	Fwd: 5’-AATGAAAAGGCCCCCAAGGTAGTTATCC-3’
MYC	Rev: 5’-GTCGTTTCCGCAACAAGTCCTCTTC-3’
BMI1	Fwd: 5’-GGTACTTCATTGATGCCACAAC-3’
BMI1	Rev: 5’-TGCTGGGCATCGTAAGTATC-3’
RING1	Fwd: 5’-CCAGCAAAACGTGGGAACTG-3’
RING1	Rev: 5’-GCAGAATCTGTGGAGGCACT-3’
RING2	Fwd: 5’-CTAAGGCCAGACCCAAACTT-3’
RING2	Rev: 5’-TGCTTGTTGATCCTGGCTAATA-3’
PTEN	Fwd: 5’-AAGGGACGAACTGGTGTAATG-3’
PTEN	Rev: 5’-GCCTCTGACTGGGAATAGTTA-3’
p14ARF	Fwd: 5’-GGTCGGGTAGAGGAGGT-3’
p14ARF	Rev: 5’-CCCATCATCATGACCTGGAT-3’
BIM	Fwd: 5’-CAGGCCTTCAACCACTATCTC-3’
BIM	Rev: 5’-AACTCTTGGGCGATCCATATC-3’
β-actin	Fwd: 5’-TTCTACAATGAGCTGCGTGTG-3’
β-actin	Rev: 5’-GGGGTGTTGAAGGTCTCAAA-3’

Supplementary Table 2: Primers used for ChIP qRT-PCR.

BMI1	Fwd: 5’-GGCCTGACTACACCGACACT-3’
BMI1	Rev: 5’-GCTGAAGGCAGAGTGGAAAC-3’
RING1	Fwd: 5’-TCTTGTGTATGTCCCTGTTTCC-3’
RING1	Rev: 5’-AGCCATAGACTCGAGAATTGC-3’
RING2	Fwd: 5’-GGCCAAGGAGGTAGGAATAAAG-3’
RING2	Rev: 5’-AGTGTAATTGCTCCTGGGAAA-3’
GAPDH	Fwd: 5’-TACTAGCGGTTTTACGGGCG-3’
GAPDH	Rev: 5’-TCGAACAGGAGGAGCAGAGAGCGA-3’
